# Design of Chitosan and Alginate Emulsion-Based Formulations for the Production of Monolayer Crosslinked Edible Films and Coatings

**DOI:** 10.3390/foods10071654

**Published:** 2021-07-17

**Authors:** Tiago M. Vieira, Margarida Moldão-Martins, Vítor D. Alves

**Affiliations:** LEAF, Linking, Landscape, Environment, Agriculture and Food Research centre, Instituto Superior de Agronomia, Universidade de Lisboa, Tapada da Ajuda, 1349-017 Lisboa, Portugal; tdmvieira@gmail.com (T.M.V.); mmoldao@isa.ulisboa.pt (M.M.-M.)

**Keywords:** chitosan, sodium alginate, emulsion edible films, crosslinking, water barrier, biodegradable packaging

## Abstract

This study aimed to develop edible monolayer emulsion-based barriers with polysaccharides as film-forming components (chitosan and sodium alginate), soy lecithin as a surfactant and olive oil as a hydrophobic barrier. Monolayer barriers in the form of films were prepared by casting filmogenic emulsions composed of 2% *w*/*v* chitosan (dissolved in lactic acid 1% *v*/*v*) or 1% *w*/*v* sodium alginate, with different lipid contents (25, 50 and 100% *w*/*w* biopolymer basis) and different surfactant concentrations (5, 10 and 25% *w*/*w*, lipid basis). Glycerol was used as a plasticizer (25 % *w*/*w*, biopolymer basis). After the emulsion drying process, the obtained stand-alone films were sprayed with a crosslinking solution, achieving an optimized crosslinker content of 3.2 mgCa^2+^/cm^2^ alginate film and 4 mg tripolyphosphate/cm^2^ chitosan film. The effect of oil and lecithin contents, as well the presence of crosslinking agents, on the film’s water vapour permeability (WVP), water vapour sorption capacity, mechanical properties and colour parameters, was evaluated. The results have shown that the lowest WVP values were obtained with formulations containing 25% lipid and 25% surfactant for chitosan films, and 100% lipid and 25% surfactant for alginate films. The application of the crosslinking agents decreased even further the WVP, especially for chitosan films (by 30%). Crosslinking also increased films’ resistance to deformation under tensile tests. Overall, the films developed present a good potential as polysaccharide-based barriers with increased resistance to water, which envisages the use of the designed formulations to produce either edible/biodegradable films or edible coatings.

## 1. Introduction

Packaging plays an important role in food preservation and the materials used are mainly petroleum-based polymers. Due to the negative environmental impact caused by these polymers, research has been focused on applying biodegradable polymers to the development of environmentally friendly packaging strategies. One of the most studied has been the development of biodegradable films and edible coatings. Biopolymers (e.g., polysaccharides and proteins) have been extensively studied for this purpose and applied in the preservation of a wide range of food products, such as fruits and vegetables, meat and fish products, nuts and cheese [1,2,3,4,5,6]. Polysaccharide films have a low permeability to gases at low relative humidity conditions and good mechanical properties [7,8].

Chitosan is a cationic polysaccharide extracted from chitin, an abundant constituent in the exoskeleton of crustaceans [8,9], the second most abundant polysaccharide resource on the planet after cellulose [8]. Several factors, make it particularly suitable for the formulation of edible films or coating, such as its biocompatibility, biodegradability, non-toxic, antimicrobial activity [10] and the ability to form transparent and resistant films. Moreover, plasticizers are usually applied to improve edible films or coatings flexibility, and crosslinkers are used to limit their solubility and improve mechanical strength [9]. Blended films or coatings of chitosan with other biopolymers have been fabricated using solution-casting, layer-by-layer, extrusion and other techniques, and studied for their physicochemical, functional and antimicrobial properties for application as food packaging materials. Chitosan composites with numerous natural antioxidants and antimicrobial components (e.g., plant/fruit extracts, essential oils and other phytochemicals), and Generally Recognized as Safe (GRAS) nanomaterials have also attracted significant research focus in recent years [11].

Alternatively, alginate is one of the most frequently used polymer among all seaweed polysaccharides for packaging materials [12]. It is derived from brown algae extracts, is a natural ionic polysaccharide giving rise to a chain–chain association [13], and due to its distinctive colloidal characteristics, such as thickening, gel-forming and emulsion stabilizing agent, has great interest as an effective biopolymer film or coating constituent [8,14,15]. Recent advances in the field of alginate-based composites with other synthetic polymers (e.g., PVA), nanofillers (e.g., CNF, ZnO or SiO_2_) and other biopolymers (e.g., cellulose, chitosan) for biodegradable green packaging materials, have shown the improvement in tensile, water barrier and thermal properties; along with their antimicrobial properties (e.g., with the addition of plant extracts, essential oils), enabling their usage in food packaging [12]. Polymeric structures based on alginate are very soluble in water. The crosslinking with several divalent cations can improve the characteristics of these materials, such as moisture resistance, mechanical strength, barrier properties, cohesion and stiffness [14]. 

To overcome the fact hydrocolloids such as chitosan or alginate, for being hydrophilic materials, produce poor barriers to water limiting the range of their applications, besides crosslinking, they are usually combined with lipids to form bilayer or emulsion films. Different lipids (fats and oils) have been incorporated into film-forming solutions to form the emulsion-based structure. However, among them, animal fats and plant waxes, vegetable oils and fatty acids are the most popular [16]. Properties of emulsion based-films depend on the nature of the lipids, the chain length of the fatty acids and the structure of the dried emulsion which constitutes the film; moreover, the functional properties of lipid-based edible films are partially explained by both lipid nature (polarity) and structure (solid fat content, crystal type). The hydrophobic characteristic of solid lipids forms thicker and more brittle films [17]. Vegetable oils (corn oil, olive oil, rapeseed oil, sunflower oil) are easily available, low cost, non-toxic, non-depletable and non-volatile; furthermore, are a source of monounsaturated fatty acids, and their incorporation as edible coating to food products is associated with various positive health benefits [16]. Some authors have reported composite films featuring unsaturated oils that can potentially improve the moisture-barrier properties of hydrophilic films, preventing drastic changes in the mechanical properties of the emulsified films, as these are liquid at room temperature [6,9,18,19,20]. Emulsifiers such as lecithins have often been added to emulsion film and coating formulations to improve their functional characteristics by stabilizing dispersed systems in composite emulsion-based edible films [16].

The goal of this work was to develop edible emulsion-based barriers in the form of films, with polysaccharides as film-forming components (chitosan and sodium alginate) and olive oil as a hydrophobic barrier. A stepwise approach was used to define the most suitable plasticizer, olive oil, surfactant and crosslinker content. The goal was to optimize monolayer barriers, instead of bilayers [9], in order to decrease costs associated with materials and production process. In addition, regarding the crosslinking methodology, the amount of crosslinker added was optimized by spraying the crosslinking solution instead of films’ immersion in it, as commonly used. Films’ immersion in the crosslinker solution has the drawback of losing the plasticizer by diffusion to the solution, increasing the possibility of ending with rigid structures prone to fracture. Films were characterized in terms of water vapour permeability (WVP), hygroscopic, mechanical and colour properties. It is envisaged the design of formulations that enable to produce, not only films, but also edible coatings for fruits, with enhanced water resistance. 

## 2. Materials and Methods

### 2.1. Materials

Chitosan was purchased from Golden/Shell Biochemical Co.Ltd (Zhejiang, China), sodium alginate from Quimidroga, S.A. (Barcelona, Spain), lactic acid from Panreac Quimica SAU (Barcelona, Spain) and calcium chloride supplied from Absolve ^®^ (Odivelas, Portugal). The soy lecithin was purchased from Tokyo Chemical Industry Co., LTD. (Tokyo, Japan), and olive oil (Lisbon, Portugal) was acquired at a local store.

### 2.2. Films Preparation

A chitosan concentration of 2% *w*/*v* (dissolved in lactic acid 1% *v*/*v*) and 1% *w*/*v* sodium alginate (dissolved in deionized water) were selected according to several studies referred in the literature, namely from Reyes-Avalos et al. work [9], and monolayers of chitosan/olive oil and alginate/olive emulsion edible films were prepared separately. To select the plasticizer concentration (glycerol), different plasticizer contents were added (25, 50 and 100% biopolymer basis) into each polymer solution. The formulations were transferred to plastic Petri dishes and dried at room temperature (22 °C) for 72 h to form monolayer films. The ones producing films without ruptures or brittle zones and with good mechanical resistance when handled were selected and used in the next step. Monolayer films were then further optimized using the formulations presented in Table 1.

Filmogenic emulsions composed of 2% *w*/*v* chitosan (dissolved in lactic acid 1% *v*/*v*) or 1% *w*/*v* sodium alginate, with different lipid contents (olive oil: 25, 50 and 100% *w*/*w* biopolymer basis) and different surfactant concentrations (soy lecithin: 5, 10 and 25% *w*/*w* lipid basis) were tested. Glycerol was used at a concentration previously selected (25% *w*/*w* biopolymer basis for chitosan films and 50% *w*/*w* for sodium alginate-based films). The mixtures were homogenized at 13,500 rpm for 2 min using an Ultraturrax T25 basic (IKA^®^ Works, Inc., Wilmington, NC, USA). Then emulsions were transferred to plastic Petri dishes and dried at room temperature (22 °C) for 72 h. 

After the emulsion drying process, the obtained stand-alone films were sprayed with a crosslinking solution (calcium chloride 6% *w*/*v* for sodium alginate-based films and sodium tripolyphosphate 6% *w*/*v* for chitosan-based films). To evaluate the most suitable crosslinker amount, a content from 0 to 32 mg/cm^2^ dry film was applied, followed by drying overnight at room temperature. Finally, all film samples were stored for 48 h at ambient temperature and 55% RH before being characterized.

### 2.3. Films’ Characterization

#### 2.3.1. Thickness

The film’s thickness was measured with a hand-held digital micrometer (Digimatic Micrometer, Mitutoyo, Japan). Measurements were carried out at four positions in the samples used for measuring water vapour permeability, and in two positions along the rectangular strips used for the mechanical properties’ evaluation. Mean values were used in the calculations.

#### 2.3.2. Water Vapour Permeability (WVP)

The gravimetric method was employed to measure WVP based on Alves, et al. [21]. Each film sample without defects was sealed with silicone on the top of a cylindrical glass permeation cell (inner diameter = 50 mm, height = 13 mm) that was placed in a desiccator. To maintain a 40% relative humidity (RH) gradient across the film, a saturated KNO_3_ solution (a_w_ = 0.936) was placed inside the cell and a saturated Mg (NO_3_)_2_·6H_2_O solution (a_w_ = 0.534) was added into the desiccator. A fan was used to promote the circulation of air inside the desiccator and minimize the mass transfer resistance of the air boundary layer above the film. The room temperature and the RH outside the permeation cell were measured over time using a thermohygrometer (Vaisala, Finland). The water vapour molar flux (*Nw*) was determined from the weight loss of the permeation cell, measured at regular time intervals for 8 h. The measurements were carried out in triplicate. The water vapour permeability was calculated using Equation (1):

(1) $$WVP=\frac{Nw\times \delta \;}{\Delta Pw,\;eff}$$

where δ is the film thickness and Δ*P_w,eff_* is the effective driving force, expressed as the water vapour pressure difference between both sides of the film [21]. Films with lower WVP were selected for further characterization.

#### 2.3.3. Water Absorption Capacity and Solubility in Water

To determine the resistance to liquid water of selected films, the water absorption capacity (WAC) and solubility in water (SW), were measured in triplicate based on ASTM ASTM D5229 / D5229M-14e1 (2014) protocol [22]. Samples (25 × 25 mm) were dried for 24 h at 40 ± 2 °C. After weighed, the dried film samples were immersed in distilled water (35 mL) for 48h at room temperature. After this period, the water excess was removed from the surface of each film sample, and it was weighted again. The value of WAC was calculated with the following equation:

(2) $$WAC\;\left(\%\right)=\frac{mf-mi}{mi}\times 100$$

where *mf* and *mi* are the film sample mass (g) after and before immersion, respectively. Subsequently, the film samples after immersion were dried at 70 °C for 24 h, after which were weighted again (*mfd*). The SW value was calculated by:

(3) $$SW\;\left(\%\right)=\frac{mi-mfd}{mi}\times 100$$

#### 2.3.4. Water Vapour Sorption Isotherms

The water vapour sorption isotherms of selected films were determined by a gravimetric method, using desiccators containing saturated salt solutions: LiCl, CH_3_COOK, CaCl_2_, K_2_CO_3_, Mg(NO_3_)_2_.6H_2_O, NaNO_2_, NaCl, KCl, BaCl_2_ with a water activity at 20 °C of 0.122, 0.231, 0.323, 0.438, 0.547, 0.660, 0.757, 0.854, 0.91, respectively [23]. Film samples (25 × 25 mm) were dried at 40 °C for 48 h and weighed to determine their dry weight. Then, samples were stabilized at room temperature in desiccators containing the saturated salt solutions, until reaching constant weight. The equilibrium moisture content was calculated as the difference between the mass of the sample at equilibrium and the sample dry mass, divided by the dry mass. Three replicates of each film were tested.

The Peleg model (Equation (4)) [24] was fitted to experimental data:

*Xe* = A*a_w_^B^ + C*a_w_^D^ (4)

where *Xe* is equilibrium moisture (g water/ g dry film), *a_w_* is the water activity and *A*, *B*, *C* are the Peleg model constants. The goodness of the fit was assessed based on the r^2^ value, the standard deviation of the estimate (SDE) and mean relative error (MRE) [25], calculated as following equations:

(5) $$\mathrm{SDE}=\sqrt{\frac{\sum_{i=1}^{n}\left[Y-\hat{Y}\right]}{DF}}$$

(6) $$\mathrm{MRE}=\frac{100}{n}\sum_{i=1}^{n}\left(\frac{\left|Y-\hat{Y}\right|}{Y}\right)$$

In which, *n* is the number of experimental observations, Y is the experimentally observed values of equilibrium moisture content; Ŷ is the value of equilibrium moisture content calculated by the model; *DF* refers to the degree of freedom of the model.

#### 2.3.5. Mechanical Properties

The tensile strength (TS) and elongation at break (EB) of selected films (strips 70 × 25 mm^2^) were determined under tensile testes using a Texturometer (TA-XT2, Stable Micro System, UK). Before mechanical testing, samples were conditioned at 25 °C and 55% RH for 48 h. Samples were fixed by tensile grips and force and deformation were recorded during extension at a constant velocity of 0.5 mm/s, with an initial distance between the grips of 50 mm. TS (maximum force/initial cross-sectional area) and EB (film elongation at rupture/initial gauge length) were determined directly from the stress-strain curves. The Young’s modulus (YM), expressed in MPa, was calculated as the slope of the elastic region of the stress-strain curves, according to ASTM D882-18 (2018) methods [26]. At least seven replicates for each film formulation were analyzed.

#### 2.3.6. Optical Properties

The colour alterations on objects caused by the application of selected films were evaluated by measuring the colour parameters of coloured paper sheets, covered and uncovered by the test films. A colourimeter (Konica Minolta CTR-300, Williams Drive Ramsey, NJ, USA) was used with the CIEL*a*b* colour space. The colourimeter was calibrated with a white standard (L* = 94.62; a* = −0.53 and b* = 3.64, where L* indicates lightness and a* and b* are chromaticity coordinates: a* from red to green and b* from blue to yellow). The colour differences (ΔE) were calculated by:

(7) $$\Delta E=\sqrt{{\left(L_{0}^{*}-L_{}^{*}\right)}^{2}+{\left(a_{0}^{*}-a_{}^{*}\right)}^{2}+{\left(b_{0}^{*}-b_{}^{*}\right)}^{2}}$$

where subscript 0 refers to uncovered coloured paper sheets. Five measurements on different areas of the coloured sheets with and without films were performed.

### 2.4. Statistical Analysis

Statistica 8.0 software (Statsoft Inc., Tulsa, OK, USA) was used for statistical analysis. Analysis of variance (ANOVA) and Tukey’s multiple range test (p level of 0.05) to detect differences among mean values of film’s properties was used. Model fitting was performed using OriginPro 8.0 software (OriginLab, Northampton, MA, USA).

## 3. Results and Discussion

### 3.1. Films Properties When Handled

To select the most suitable amount of glycerol used as a plasticizer, films were produced with chitosan (2% *w*/*v*, dissolved in lactic acid 1% *v*/*v*) and sodium alginate (1% *w*/*v*, dissolved in deionized water) with different plasticizer contents (25, 50 and 100% *w*/*w* biopolymer basis). Films were analyzed in terms of appearance and mechanical properties when handled. It was observed that, for chitosan films, a glycerol concentration equal to or greater than 50% *w*/*w* originated films with inadequate mechanical properties, presenting an excessive deformation, high adhesion and risk of rupture. Similar properties were observed for sodium alginate films with a glycerol concentration of 100% *w*/*w*. Conversely, good mechanical properties with negligible adhesion were observed for chitosan and alginate films with 25 and 50% *w*/*w* glycerol content, respectively. These formulations were selected for the next studies. 

The emulsion-based films were prepared according to the formulations presented in Table 1 and have shown to be flexible, easy to handle and the ability to bend without breaking. A non-significant olive oil exudate was detected, indicating good and stable oil incorporation within the film’s matrix. The control films without olive oil were transparent, while those containing oil were less transparent and opaque. Similar observations were referred to in the literature for biopolymer films containing lipids [27,28].

### 3.2. Water Vapour Permeability

**Figure 1:**
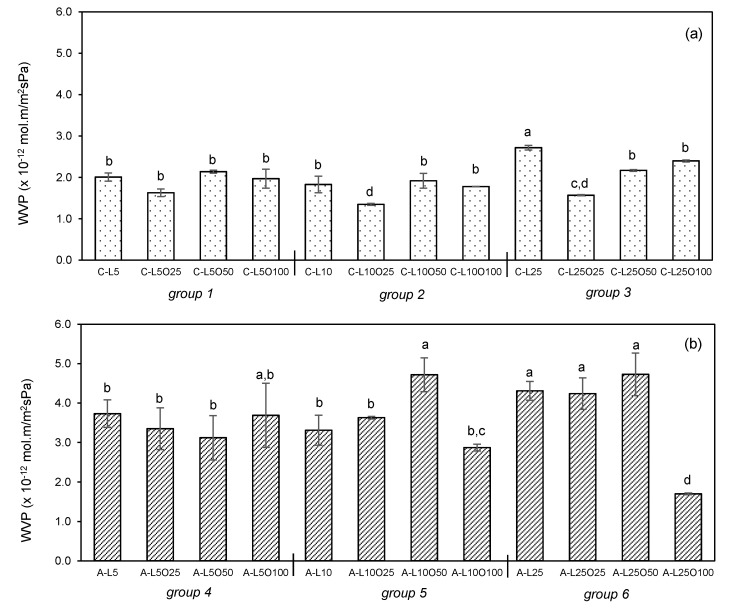
Water vapour permeability (WVP) of (**a**) chitosan and (**b**) alginate-based films, varying the olive oil (O, % *w*/*w*) and soybean lecithin (L, % *w*/*w*) contents according to Table 1. Bars are expressed as mean ± standard deviation. Means in bars with different letters are significantly different (*p* < 0.05), based on Tukey’s test performed separately for alginate and chitosan-based films.

Edible films may be used as a barrier to moisture transfer between food and the surrounding atmosphere preventing dehydration, which can induce food physicochemical and biochemical changes during storage. To enhance the water barrier properties of carbohydrate films, the incorporation of edible oils in the polymer matrix has been studied. In this section, a first screening was performed for the selection of the film formulations presented in Table 1 that enable the production of films with the lowest WVP. The results are shown in Figure 1.

The WVP values varied substantially among emulsion alginate-based films samples (1.70–4.73 × 10^−12^ mol.m/m^2^.s.Pa). A significantly lower WVP was observed for films with 100% (*w*/*w*) oil and 25% (*w*/*w*) lecithin (A-L25O100) when compared to all the other formulations. This means that films resulting from emulsions prepared with alginate solutions as a continuous phase needed a high oil content to demonstrate enhanced barrier properties to water vapour. Chitosan-based films showed higher water vapour barrier properties for all formulations, with WVP values around half of those observed for alginate-based films (1.35–2.72 × 10^−12^ mol.m/m^2^.s.Pa). This fact may be attributed to the less hydrophilic nature of chitosan. Water vapour transfer depends on the hydrophilic/hydrophobic ratio of the film’s components and occurs only through the hydrophilic portion of the film [29]. Furthermore, for this group of films, significantly lower WVP values were obtained only for the filmogenic solutions C-L10O25 and C-L25O25, meaning that oil incorporation in the chitosan matrix was only effective on decreasing WVP for 10 and 25% (*w*/*w*) lecithin contents and oil content of 25% (*w*/*w*). 

The barrier properties of emulsion-based films are quite dependent on the size, number and distribution of oil droplets in the polymeric matrix. These parameters result from the conjugation of several factors, such as biopolymers and surfactant chemical-physical properties and concentrations, the viscosity of the continuous phase and energy input during the emulsification process [30,31]. In addition, the drying step conditions (e.g., temperature, time) after emulsion casting are also important as they may promote droplets coalescence and phase separation. In this work, although the drying conditions were the same for all films, the other factors ended up producing films with different internal structures. In the specific case of chitosan-based films, a higher tendency for oil droplets coalescence may be the reason for the WPV increase for oil content above 25% (*w*/*w*), for which higher droplets in a lower number are envisaged to be present resulting in favourable conditions for an increased water diffusion rate through the films. Results of improved barrier properties by incorporation of lipids into the edible films by an emulsification process were reported by several authors, such as combining sunflower oil with quinoa/chitosan (WVP from 1.8–2.6 × 10^−13^ mol.m/m^2^.s.Pa) [20] and starch (WVP from 0.5–1.1 × 10^−11^ mol.m/m^2^.s.Pa) [32]; oleic acid with carboxymethyl cellulose (WVP from 0.4–1.5 × 10^−11^ mol.m/m^2^.s.Pa) [18] and kefiran (WVP of 2.2 × 10^−12^ mol.m/m^2^.s.Pa) [19], as well as palmic and stearic acids combined with corn starch (WVP from 0.6–7.2 × 10^−10^ mol.m/m^2^.s.Pa) [33]. The variability of WVP values between works is not only due to the different materials used. Due to the hydrophilic nature of the biopolymers, the WVP value is highly dependent on the driving force applied in the test (relative humidity difference between both sides of the film), on the relative humidity value at which the films were equilibrated before measurement and on the films thickness. These facts turn quite difficult to isolate the effect of type of oil between films from different works. Still, it was observed that in each work, the inclusion of oils in the polymeric matrix decreased the WPV value when it was measured in the same conditions.

From the results obtained, the films presenting the lower WVP (A-L10O100, A-L25O100, C-L10O25 and C-L25O25) were selected for the crosslinking step and further characterization.

### 3.3. Films’ Crosslinking

Resistance of edible films to water is desirable if the film is to be used for the preservation of intermediate and high-moisture food products, or to the stored under high relative humidity conditions. Even though the water resistance is expected to be higher for emulsion-based films, imparted by the oil droplets, it may be further increased by crosslinking reactions within the polymeric matrix. 

Crosslinking of dried emulsion films was performed by spraying aqueous solutions of crosslinking agents on the film’s surface (calcium chloride and sodium tripolyphosphate for alginate and chitosan-based films, respectively). Crosslinking takes place by establishing ionic bonds between alginate chains and Ca^2+^ ions, and between TPP ions and chitosan chains [34,35].

**Figure 2:**
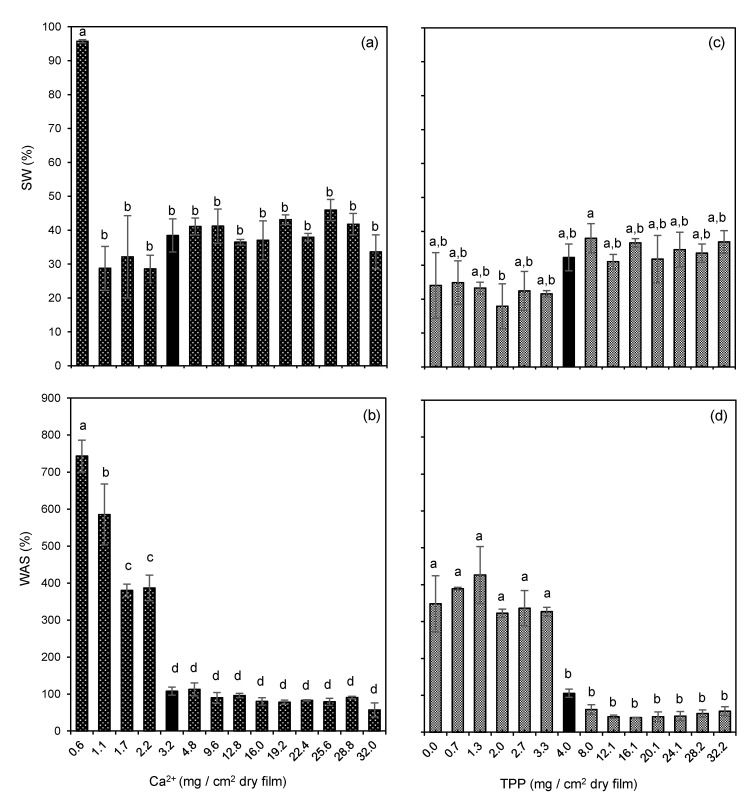
Influence of crosslinker content sprayed on film’s surface on their solubility in water (SW) and water absorption capacity (WAC). (**a**,**b**) refer to the alginate-emulsion films A-L25O100; (**c**,**d**) refer to the chitosan-emulsion films C-L25O25. Bars indicate the mean ± standard deviation. Means in bars with different letters are significantly different (*p* < 0.05), based on Tukey’s test, separately for each data set from a) to d). Bars in black refer to crosslinking mass selected for the next steps of this study.

The effect of different amounts of crosslinkers was assessed by measuring films’ water absorption capacity (WAC) and solubility in water (SW). This effect was quite similar among all chitosan and alginate-based films prepared according to Table 1. As examples, Figure 2 presents the variation of WAC and SW with increasing crosslinking content for C-L25O25 and A-L25O100 films.

It may be observed that alginate-based films are readily disintegrated for the lowest mass of crosslinker applied (0.6 mg/cm^2^ dry film), due to the high solubility in water of alginate and to the insufficient calcium ions added to maintain a stable reticulated network. However, for a crosslinker addition equal to or above 1.1 mg/cm^2^ dry film, SW decreased markedly to around 30% and did not change significantly with the increase of crosslinker mass added. This fact is attributed to the formation of enough calcium bridges to maintain the polymeric matrix, being most of the film’s SW a consequence of the diffusion of low molecular weight compounds, such as glycerol and free lecithin, to water. On the contrary, chitosan-based films presented non statistically different SW values (from 26% to around 40%), including when no crosslinker was added. These results are explained by the low solubility of chitosan itself in water at pH > 4. Similarly, the observed SW values are also mainly attributed to the transfer of glycerol and free lecithin molecules from the films’ matrix to the surrounding water. 

In what concerns WAC, it is observed a particularly high value (740%) for non-disintegrated fragments of alginate-based films with 1.1 mg Ca^2+^/cm^2^ dry film, which decreased gradually until 100% with increasing crosslinker mass up to 3.2 mg Ca^2+^/cm^2^ dry film. After that, it remained statistically independent on the crosslinker amount added. Chitosan-based films presented WAC values around 350% for a TPP mass equal to or below 3.3 mg/cm^2^ dry film, with a sudden decrease to 100% for 4 mg TPP/cm^2^ dry film and above. A higher crosslinking degree leads to a stiffer polymer network with lower molecule mobility, with lower intermolecular spaces to accommodate water molecules. From the results, we may infer about the amount of crosslinker needed to bind to most of the active sites in the polymeric matrices (3.2 mg Ca^2+^ and 4 mg TPP/cm^2^ dry film, for alginate and chitosan-based films, respectively), above which additional calcium and TPP ions did not increase the crosslinking degree resulting in unchanged WAC values.

### 3.4. Characterization of Selected Films

Based on the results of Section 3.2, films presenting a lower water vapour permeability (C-L10O25, C-L25O25, A-L10O100 and A-L25O100) and crosslinked under the most favourable conditions obtained in Section 3.3 (3.2 mg Ca^2+^ and 4 mg TPP/cm^2^ dry film, for alginate and chitosan-based films, respectively), were select for further characterization.

#### 3.4.1. Water Vapour Permeability

**Figure 3:**
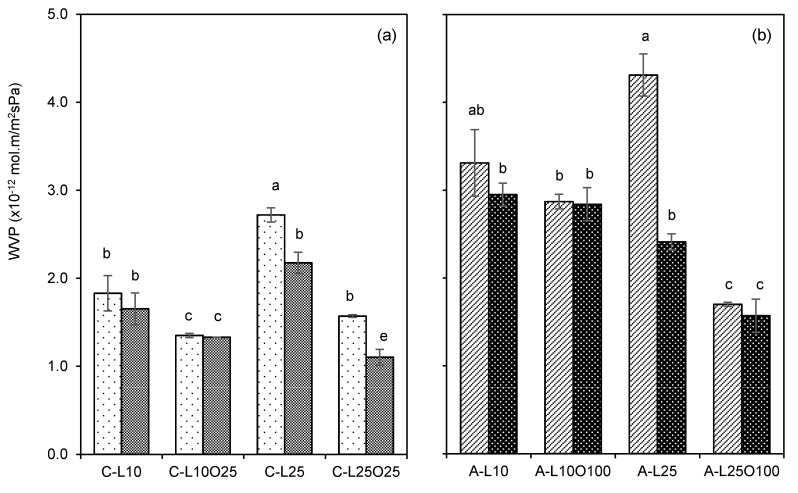
Influence of oil and crosslinking on films water vapour permeability (WVP). (**a**) refers to the chitosan-based films and (**b**) to alginate-based films, varying the olive oil (O, % *w*/*w*) and soybean lecithin (L, % *w*/*w*) contents according to Table 1. Lighter and darker bars refer to non-crosslinked and crosslinked films, respectively. Bars indicate the mean ± standard deviation. Means in bars with different letters are significantly different (*p* < 0.05), based on Tukey’s test.

Figure 3 presents the water vapour permeability (WVP) values for uncrosslinked and crosslinked selected films (C-L10O25, C-L25O25, A-L10O100 and A-L25O100), along with that of their uncrosslinked and crosslinked counterparts without oil (C-L10, C-L25, A-L10 and A-L25), to separate the effects of oil and crosslinking on WVP. 

The inclusion of 25% oil in the chitosan-based films without crosslinking led to a decrease of WVP for both contents of lecithin studied (Figure 3a). When crosslinking was applied, a further significant WVP decrease (by 30%) was detected, but only for C-L25O25 films. Considering the chemical structure of soy lecithin, presenting a positively charged choline chemical group in the polar region of the molecule, we may envisage a participation of this chemical group in crosslinking reactions with the negatively charged tripolyphosphate. In this way, a denser crosslinked network (chitosan-tripolyphosphate-choline group) may cause a lower water diffusion coefficient, and consequently a lower WVP, only detected for films with higher lecithin content. In what concerns alginate-based films, the inclusion of oil without crosslinking also decreased WVP, and much more significantly when content of 25% lecithin was used (A-L25 and A-L25O100) (Figure 3b). However, the barrier properties of A-L25O100 emulsion films were not significantly improved with the application of crosslinking, and the WVP reduction is attributed only to the presence of oil droplets.

#### 3.4.2. Water Vapour Sorption Isotherms

**Figure 4:**
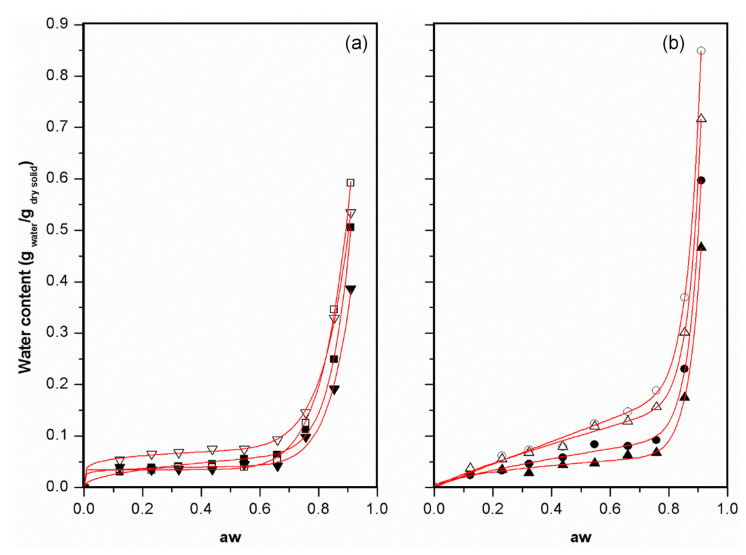
Equilibrium moisture sorption isotherms of selected films fitted to Peleg model: (**a**) Chitosan (C) based films: 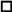 C-L10 and 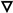 C-L25 (crosslinked chitosan-based films without olive oil); 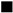 C-L10O25 and 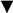 C-L25O25 (crosslinked chitosan-based emulsion films); (**b**) Alginate (A) based films: 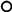 A- L10 and 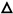 A-L25 (crosslinked alginate-based films without olive oil); 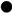 A-L10O100 and 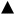 A-L25O100 (crosslinked alginate-based emulsion films). Films contain different contents of soybean lecithin (L, %*w*/*w* lipid basis) and olive oil (O, %*w*/*w* polymer basis).

Water vapour sorption isotherms of crosslinked chitosan and alginate-based films are presented in Figure 4. For all films, a variation in adsorbed water was small for water activity up to 0.75, but for higher values between 0.75 and 0.91, there was a sharp increase in the amount of water adsorbed, which was also observed by other researchers for polysaccharide-based films and particles [5,36]. The water vapour sorption isotherms followed typical type III behaviour according to Brunauer et al. [37]. The shape of all curves is common for high carbohydrate content products, which absorb a relatively small amount of water at low water activities and a large amount at high water activity values.

Peleg model was fitted to water vapour sorption data. The high determination coefficient (>0.986), low value of mean relative error (4.2–8.0%) and standard deviation of the estimate (0.014–0.025), confirm a good concordance of that model with experimental data (Table 2).

Crosslinked chitosan and alginate-based films without oil (C-L10, C-L25, A- L10 and A-L25) were found to be more sensitive to environmental humidity than crosslinked chitosan and alginate-based films with oil, for the a_w_ range studied. The presence of oil decreased considerably the equilibrium moisture content on higher aw (0.91) within the chitosan films samples by 16.7% and 27.3% (C-L10O25 and C-L25O25) (Figure 4a), and also within the alginate-based films samples by 33.3% and 35.7% (A-L10O100 and A-L25O100) (Figure 4b), when compared to the films with no oil added. Sorption isotherms with lower equilibrium moisture content caused by the presence of different lipids were observed, such as in caseinate/oleic acid films [38], pullulan/rice wax films [39], agar or cassava starch films with an hydrogenated vegetable oil [40] and alginate or carrageenan films with a blend of acetic acid esters of mono and diglycerides mixed with 20% *w*/*w* beeswax [41]. The incorporation of lipids reduces moisture sorption because, due to their hydrophobic nature, they correspond to a fraction of film with quite low water uptake capacity [38].

#### 3.4.3. Optical Properties

Since the colour of edible films may affect consumer acceptance, it is of primary importance that its transparency is preserved or at least they display colour as close as possible to the natural pigment of foods on which the film is going to be applied [42]. Colour difference (∆E) was analyzed, as it provides a good analysis of film colour attributes because it includes the three-colour parameters: lightness (L*), red-green (a*) and yellow-blue (b*) coordinates [43]. Table 3 shows the values of colour alteration of objects due to the application of the films by measuring the colour parameters of coloured paper sheets, uncovered and covered by the film samples.

The inclusion of olive oil in the structure of both chitosan and alginate-based films induced non-significant colour alterations. The exception was when films were applied over a white colour, for which a higher ∆E was caused by emulsion films when compared to their counterparts without oil. From a visual inspection, all films were transparent, but those with olive oil tended to show a light yellowish colour, which is more easily perceived over a white background. Crosslinking reactions also did affect colour, as crosslinked emulsion films tended to show higher ∆E values compared to non-crosslinked ones. Nevertheless, the alteration of the colour induced by all films were in general low (<15.3), but with values representing colour changes that may be perceived by the human eye. Among the colours studied, green seems to be the one less affected by films application showing ∆E values below 8.1, close to the value generally accepted for food products (∆E = 6).

According to basic colour measurement principles, the amount of light reflected depends partially on the amount absorbed, the amount penetrating through the sample and the amount reflected by any background used behind the sample [44]. Given this, the thickness can affect the light reflected and consecutively change colour parameters. This fact is quite relevant if the emulsion-based emulsions are intended to be applied, not only for stand-alone film’s production but also for edible coatings applied directly on foods surface. Coatings would present a much lower thickness than films, resulting in lower food surface colour alteration.

#### 3.4.4. Mechanical Properties

**Figure 5:**
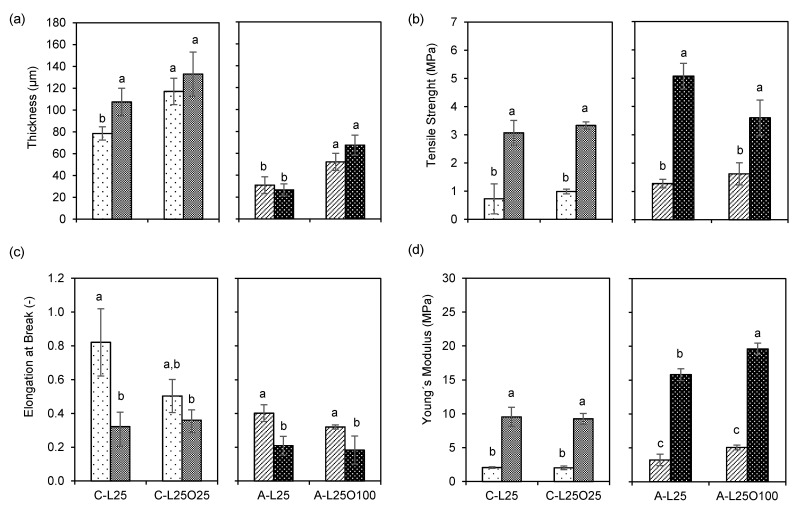
Values of the thickness (**a**), tensile strength (**b**), elongation at break (**c**) and Young’s modulus (**d**) for uncrosslinked and crosslinked selected films (C-L10O25, C-L25O25, A-L10O100 and A-L25O100), along with that of their uncrosslinked and crosslinked counterparts without oil (C-L10, C-L25, A-L10 and A-L25). Lighter and darker bars refer to non-crosslinked and crosslinked films, respectively, and indicate the mean ± standard deviation. Means in bars with different letters are significantly different (*p* < 0.05), based on Tukey’s test.

Mechanical properties are important to ensure that the film has adequate mechanical strength and integrity during transportation, handling and storage of foods wrapped with them [45]. Tensile strength (TS) indicates the maximum tensile stress that the film can sustain, Young’s modulus (YM) is a measure of the stiffness of the film and elongation at break (EB) is the maximum change in length of a test specimen before breaking [46]. Figure 5 presents the thickness and the mechanical properties under tensile tests for uncrosslinked and crosslinked selected films (C-L25O25 and A-L25O100), along with that of their uncrosslinked and crosslinked counterparts without oil (C-L25 and A-L25). The formulations C-L25O25 and A-L25O100 were selected due to their higher barrier do water vapour and lower water vapour adsorption capacity observed in Section 3.4.1 and Section 3.4.2.

The results show that, for all films tested, the mechanical properties are not significantly different between films with oil and without oil. So, chitosan and alginate-based films with only soy lecithin as surfactant and glycerol as a plasticizer, present similar mechanical properties to those also containing oil droplets. This fact indicates that the effect of oil on mechanical properties is probability being masked by the effect of the polymeric matrix with lecithin and glycerol itself. 

In the case of chitosan emulsion films with olive oil, Pereda et al. [46] have shown an increase in TS and YM up to 15 and 140 MPa, respectively, with increasing olive oil content to 15% (*w*/*w*, biopolymer basis). These values are higher than those observed in this work (Figure 5). The authors attributed their results to crosslinking between the polymer chains and the olive oil, which decreases the free volume and the molecular mobility of the polymer. This crosslinking was referred to be a result of electrostatic interactions between the positively charged amino groups of chitosan and the carboxylate function of oleic acid [47]. However, these crosslinking interactions are not likely to occur in the present work, because an emulsifier was used, separating the oil from chitosan molecules. The same is not true in the work of Pereda et al. [46], where no emulsifier was used. In what concerns works with alginate based crosslinked films, the incorporation of cinnamon bark oil (CBO) in alginate matrices resulted in stronger films as indicated by larger values of TS (16 MPa for 100% CBO, *w*/*w* biopolymer basis) when compared to films without CBO (6.5 MPa). However, after addition of SBO, the TS of such films decreased significantly (6.7 MPa for 100% CBO plus 100% SBO, *w*/*w* biopolymer basis). This effect was attributed to the increased content of droplets containing non-polar SBO forming different structures in the film matrix [48]. The later formulation is closer to that used in the present work, where crosslinked A-L25O100 films showed a TS of about 3.6 MPa. There are other references showing a reduction of tensile parameters when long-chain fatty acids are incorporated in the polymeric matrix, such as chitosan with cinnamon oils [49] and alginate with soybean oil [50]. These different behaviours can be attributed to diverse interactions formed due to different parameters, such as the type of polymers (degree of deacetylation for chitosan, mannuronic/guluronic acid ratio for alginate and average molecular weight), type and concentration of emulsifier and oil used and drying conditions.

From Figure 5, what is changing significantly the mechanical properties is the crosslinking step. There is a significant increase in TS and YM, along with a decrease of EB after crosslinker application. These results reveal stronger structures due to lower polymer chain mobility, because of bonds established between the polymer chains and the crosslinking ions.

Overall, the optimized formulations produced films with a good potential as polysaccharide/lipid-based barriers with increased resistance to water, which is especially important for application (e.g., wrapping) in several food products (e.g., fresh fish, meats and fruits) were food moisture loss is likely to occur, along with high relative humidity conditions under packaging and storage. A recent work has shown that the designed formulations present a good potential to produce edible coatings for shelf-life extension of highly perishable fruits (e.g., whole fresh figs [51]).

## 4. Conclusions

Edible emulsion-based barriers, with polysaccharides as film-forming components (chitosan and sodium alginate) and olive oil as a hydrophobic barrier, were successfully developed and characterized in the form of films. The results have shown that the incorporation of olive oil within polymeric–glycerol matrix, using lecithin as a surfactant, improved the barrier properties to water vapour and significantly reduced moisture adsorption at high a_w_ values (>0.8). The application of crosslinking significantly enhanced the film’s mechanical properties, as well as their resistance to liquid water by decreasing their solubility in water and water absorption capacity. In addition, films induced low colour changes when applied on coloured paper, especially for green colour. The optimized formulations developed present a great potential to produce wrapping films and edible coatings for highly perishable fruits.

## Figures and Tables

**Table 1:** Chitosan and alginate -based edible films’ formulations. A refers to alginate, C to chitosan, L to lecithin and O to olive oil.

Sample	Chitosan (% *w*/*v*)	Alginate (% *w*/*v*)	Glycerol (% *w*/*w*, Biopolymer Basis)	Soy Lecithin (% *w*/*w*, Lipid Basis)	Olive Oil (% *w*/*w*, Biopolymer Basis)
**Group 1**					
C-L5	2	/	25	5	0
C-L5O25	2	/	25	5	25
C-L5O50	2	/	25	5	50
C-L5O100	2	/	25	5	100
**Group 2**					
C-L10	2	/	25	10	0
C-L10O25	2	/	25	10	25
C-L10O50	2	/	25	10	50
C-L10O100	2	/	25	10	100
**Group 3**					
C-L25	2	/	25	25	0
C-L25O25	2	/	25	25	25
C-L25O50	2	/	25	25	50
C-L25O100	2	/	25	25	100
**Group 4**					
A-L5	/	1	50	5	0
A-L5O25	/	1	50	5	25
A-L5O50	/	1	50	5	50
A-L5O100	/	1	50	5	100
**Group 5**					
A-L10	/	1	50	10	0
A-L10O25	/	1	50	10	25
A-L10O50	/	1	50	10	50
A-L10O100	/	1	50	10	100
**Group 6**					
A-L25	/	1	50	25	0
A-L25O25	/	1	50	25	25
A-L25O50	/	1	50	25	50
A-L25O100	/	1	50	25	100

**Table 2:** Peleg model parameters. ^1^ Values are expressed as mean (± standard deviation). ** ×10^−14^; R^2^: Coefficient of determination; SDE: Standard deviation of the estimate; MRE: Mean relative error.

Samples	Parameters ^1^				R^2^	SDE	MRE
	A	B	C	D			
C-L10	1.38 (0.04)	9.59 (0.26)	0.03 (0.003)	0.911 (--) **	0.999	0.014	5.2
C-L10O25	1.26 (0.18)	11.6 (1.66)	0.06 (0.03)	0.32 (0.4)	0.986	0.024	5.3
C-L25	1.17 (0.19)	9.65 (1.83)	0.08 (0.03)	0.18 (0.4)	0.987	0.014	4.2
C-L25O25	0.99 (0.12)	11.22 (1.14)	0.04 (0.01)	0.100 (--) **	0.988	0.025	8.5
A-L10	0.22 (0.02)	0.97 (0.10)	4.17 (0.4)	19.71 (1.08)	0.999	0.022	8.0
A-L10O100	0.11 (0.01)	0.74 (0.11)	3.54 (0.4)	20.86 (1.11)	0.999	0.017	6.0
A-L25	0.18 (0.01)	0.84 (0.08)	4.03 (0.4)	21.15 (1.05)	0.999	0.017	6.1
A-L25O100	0.07 (0.01)	0.51 (0.12)	2.65 (0.3)	19.98 (1.07)	0.997	0.014	7.8

**Table 3:** Colour alterations of coloured paper sheets after being covered by selected films. Standard colours: white (W), red (R), yellow (Y), blue (B), green (G). ΔE Values are expressed as means (±standard deviation). Means in each column with the same letter are not significantly different according to Tukey’s test (*p* < 0.05).

Samples	ΔE				
	W	R	Y	B	G
**Without crosslinking**					
C-L25	10.62 (0.63) a,b	5.09 (0.61) c	6.61 (0.95) b	7.23 (0.36) d	3.68 (0.40) b
C-L25O25	12.92 (0.31) a,b	8.31 (0.78) a,b	9.19 (0.65) a,b	12.96 (0.38) b,c,d	5.12 (0.52) a,b
A-L25	11.54 (1.03) a,b	9.97 (0.45) a,b	11.51 (0.25) a	9.22 (0.71) c,d	6.62 (0.33) a,b
A-L25O100	13.25 (0.60) a	7.05 (1.09) b,c	10.35 (1.39) a,b	10.15 (0.14) c,d	6.01 (0.12) a,b
**With crosslinking**					
C-L25	8.74 (0.22) b	12.24 (0.57) a,b	11.99 (0.51) a	14.36 (0.48) a,b	8.09 (0.32) a
C-L25O25	14.44 (0.51) a	12.05 (0.53) a,b	12.64 (0.95) a	15.32 (0.92) a	8.15 (0.20) a
A-L25	3.74 (0.90) c	13.56 (0.38) a	12.27 (1.01) a	10.61 (0.38) c,d	7.50 (0.70) a
A-L25O100	8.73 (1.02) b	13.38 (1.09) a	12.61 (0.77) a	10.95 (0.52) b,c	7.22 (0.55) a

## Data Availability

Not applicable.
